# Bile duct carcinoma recurrence in the papillary region in a long-term survivor of hilar cholangiocarcinoma: a case report

**DOI:** 10.1186/s13256-016-1073-6

**Published:** 2016-10-26

**Authors:** D. Buchner, U. Drebber, D. H. Chang, D. L. Stippel

**Affiliations:** 1 Department of General, Visceral and Cancer Surgery, University of Cologne, Kerpenerstr. 62, 50637 Cologne, Germany; 2 Department of General Pathology and Pathological Anatomy, University of Cologne, Cologne, Germany; 3 Department of Diagnostic and Interventional Radiology, University of Cologne, Cologne, Germany

**Keywords:** Case report, Cholangiocarcinoma, Recurrence, Surgical therapy, Targeted next-generation sequencing

## Abstract

**Background:**

Because of its high rate of early recurrence and its poor prognosis, long-term survival after cholangiocarcinoma is rare; therefore, only limited information on patients surviving more than 5 years after surgical therapy is available.

**Case presentation:**

We report the case of a 57-year-old white man who developed a distal bile duct carcinoma 9 years after curative surgical therapy of intrahepatic cholangiocarcinoma. He had undergone a right lobe hemihepatectomy 11 years ago. Nine years later, he was diagnosed with a distal bile duct carcinoma and a duodenopancreatectomy was performed. On histologic examination both carcinomas revealed a tubular and papillary growth pattern with cancer-free resection margins and for both carcinomas there were no signs of lymphatic infiltration or metastatic spreading. Targeted next-generation sequencing showed an identical activating mutation pattern in both carcinomas.

**Conclusions:**

Late recurrence of cholangiocarcinoma, even anatomically distant to the primary, in long-time survivors is possible and could be caused by a distinct tumor biology. A better understanding of the individual tumor biology could help hepatologists as well as hepatobiliary and pancreatic surgeons in their daily treatment of these patients.

## Background

Cholangiocarcinomas (CCA) account for 3 % of all gastrointestinal tumors [1], with an estimated 5-year-survival rate of approximately 10 % [2, 3].

According to their anatomical localization they are classified as intrahepatic (iCCA), perihilar (pCCA), and distal (dCCA) carcinomas [4]. In a large study of 564 patients, 8 % of patients had iCCA, 50 % had pCCA, and 42 % had dCCA [5]. Whereas pCCA and dCCA arise from biliary epithelium and peribiliary glands, iCCA derive either from biliary epithelium or from hepatic progenitor cells [1].

In Western countries, common risk factors for CCA are primary sclerosing cholangitis (PSC), inflammatory bowel disease, alcohol, tobacco smoking, fatty liver disease, diabetes, cholelithiasis, and choledocholithiasis; the common risk factor for iCCAs is viral hepatitis. Furthermore, there are gene variations, which might have the potential to enhance carcinoma development [3, 6, 7].

The only available curative treatment option is surgical resection with negative margins. Depending on the localization, there are also options for neoadjuvant, adjuvant, and palliative chemotherapy, radiation, ablative treatment, and even liver transplant [8]. The rates of 5-year survival in patients with pCCA and dCCA after surgical therapy with negative margins range from 24 to 43 % and 27 % [5], respectively. Compared to that, the overall 5-year survival rate for all stages and treatment options is less than 5 % [9, 10]. Independent factors influencing long-term survival after surgery are the pathological metastases classification (pM), the ductal margin status, and the size of the primary tumor and its histological grading [11].

Due to the reported low survival rates there are only a few patients who develop a secondary CCA. If recurrence occurs, it needs to be differentiated between recurrence as metastatic disease, a locoregional relapse, probably due to residual cells after the first operation or a second primary in other parts of the biliary system due to field change or genetic predisposition [12].

## Case presentation

### Perihilar cholangiocarcinoma

#### Patient history

Eleven years ago, a 46-year-old white man presented with painless jaundice. He showed no other symptoms and had no risk factors such as high alcohol consumption or tobacco smoking. With a bodyweight of 73 kg and a height of 175 cm his body mass index (BMI) was 24.49 kg/m^2^ at the time of presentation. His mother had colorectal cancer otherwise there was no family history.

#### Diagnostics

An endosonography showed an intraluminal polypoid tumor with disintegration of all wall layers at the bifurcation of his bile duct system. The finding was confirmed by endoscopic retrograde cholangiopancreatography (ERCP). Biopsies were taken with the pathologic results coming back inconclusive.

Computed tomography showed an intrahepatic cholestasis and two hypodense lesions in segment VIII of his liver. It showed no signs of metastasis or other unknown pathologies. The analysis of serum tumor marker carcinoembryonic antigen (CEA) was negative and cancer antigen (CA) 19-9 was found to be elevated at 75 kU/l (<37 kU/l).

#### Treatment

He was treated with right liver lobectomy with resection of the bile duct directly distal of his pancreatic head, resection of the bile duct of his left liver lobe up to the first intersection, and with a regional lymphadenectomy. The reconstruction was performed by biliodigestive anastomosis with Roux-en-Y hepaticojejunostomy. A bile duct drain was inserted in the dorsal bile duct of his left lobe and closed on day 10 after contrast imaging of the anastomosis. Postoperatively, he recovered well. On the planned day of discharge he developed an aphasia leading to further diagnostics, which showed a transitoric ischemic attack of his left-brain hemisphere. He was transferred to neurology and fully recovered.

#### Pathology

A gross examination showed a right hemihepatectomy with a 4×2×2 cm polypoid tumor in his right hepatic bile duct extending into the bile duct junction (mainly intraductal growth type). The liver parenchyma was non-cirrhotic and prominent portal tracts were seen. Resection margins were free of tumor.

**Fig. 1:**
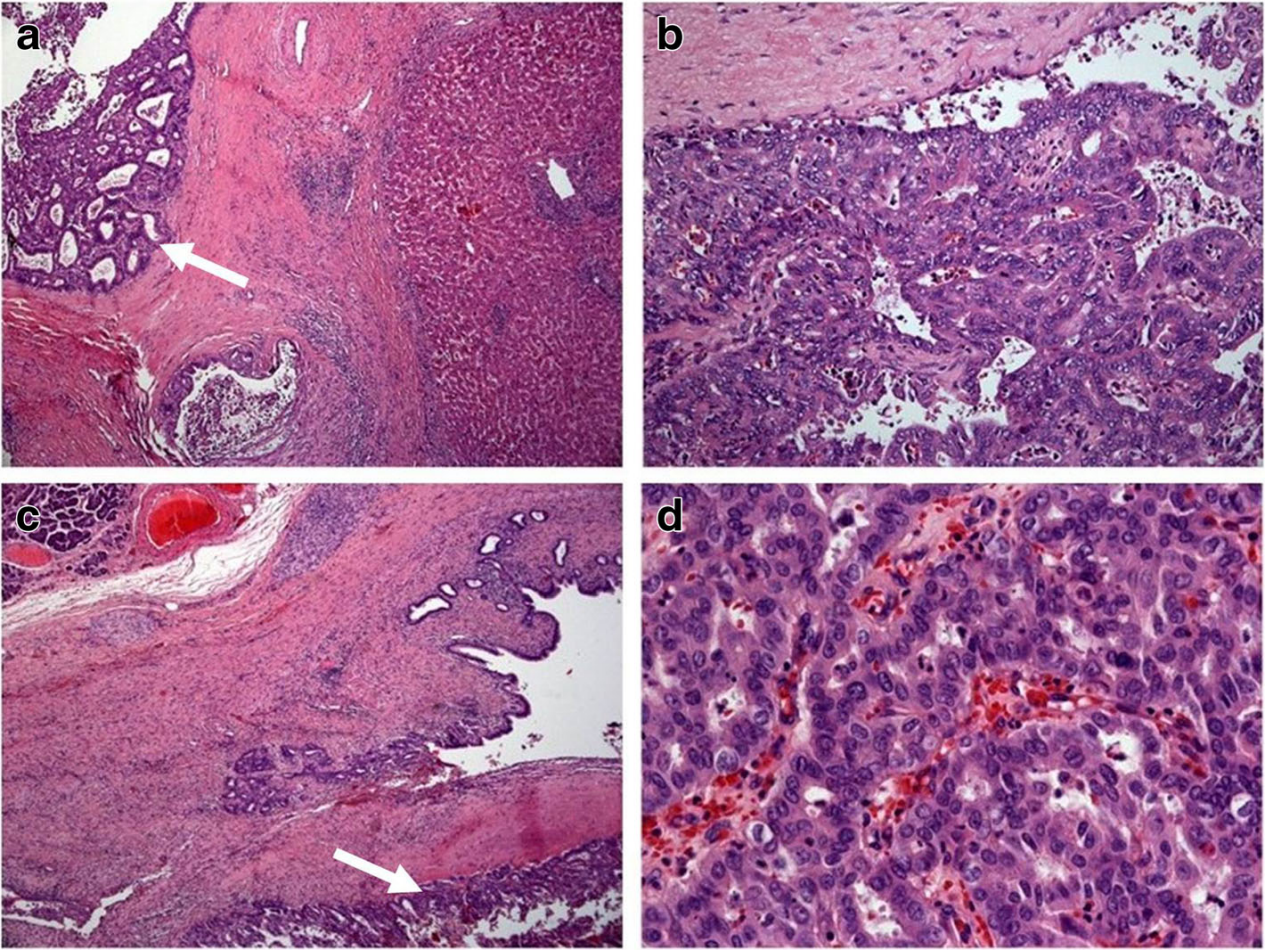
Microscopic examinations. **a, b** showing microscopic examination of the perihilar cholangiocarcinoma; **c, d** showing microscopic examination of the cholangiocarcinoma of the distal bile duct. **a** Adenocarcinoma arising from the bile duct in a large portal tract with stroma invasion. **b** On higher magnification it shows a moderately differentiated adenocarcinoma with a tubular, cribriform, and solid pattern of growth. **c** Examination of the papillary region showing pancreatic tissue in the upper left quadrant and infiltration into the papillary region by an adenocarcinoma. **d** On higher magnification it shows a poorly differentiated adenocarcinoma with tubular, cribriform, and solid growth pattern

Histologic examination revealed an intraductal tumor with tubular and papillary and solid growth pattern. There was a stromal invasion into the ductus hepaticus dexter. In the portal tracts intraductal tumor growth without stromal invasion was found (Fig. 1). According to TNM classification the tumor staging was pT1 pN0 (0/4) M0 R0, G2.

### Bile duct cholangiocarcinoma

#### Patient history

The same patient presented 2 years ago at the age of 55 with a mass of 1.8×2.4 cm at the papilla of Vater. The mass was detected via magnetic resonance imaging (MRI) during a regular cancer follow-up. In the meantime he had developed an arterial hypertension that was well controlled with ramipril 5 mg once daily. On admission he reported no weight loss, no fever, no sweating, and no signs of icterus or pruritus. His clinical examination was without any pathological finding.

#### Diagnostics

**Fig. 2:**
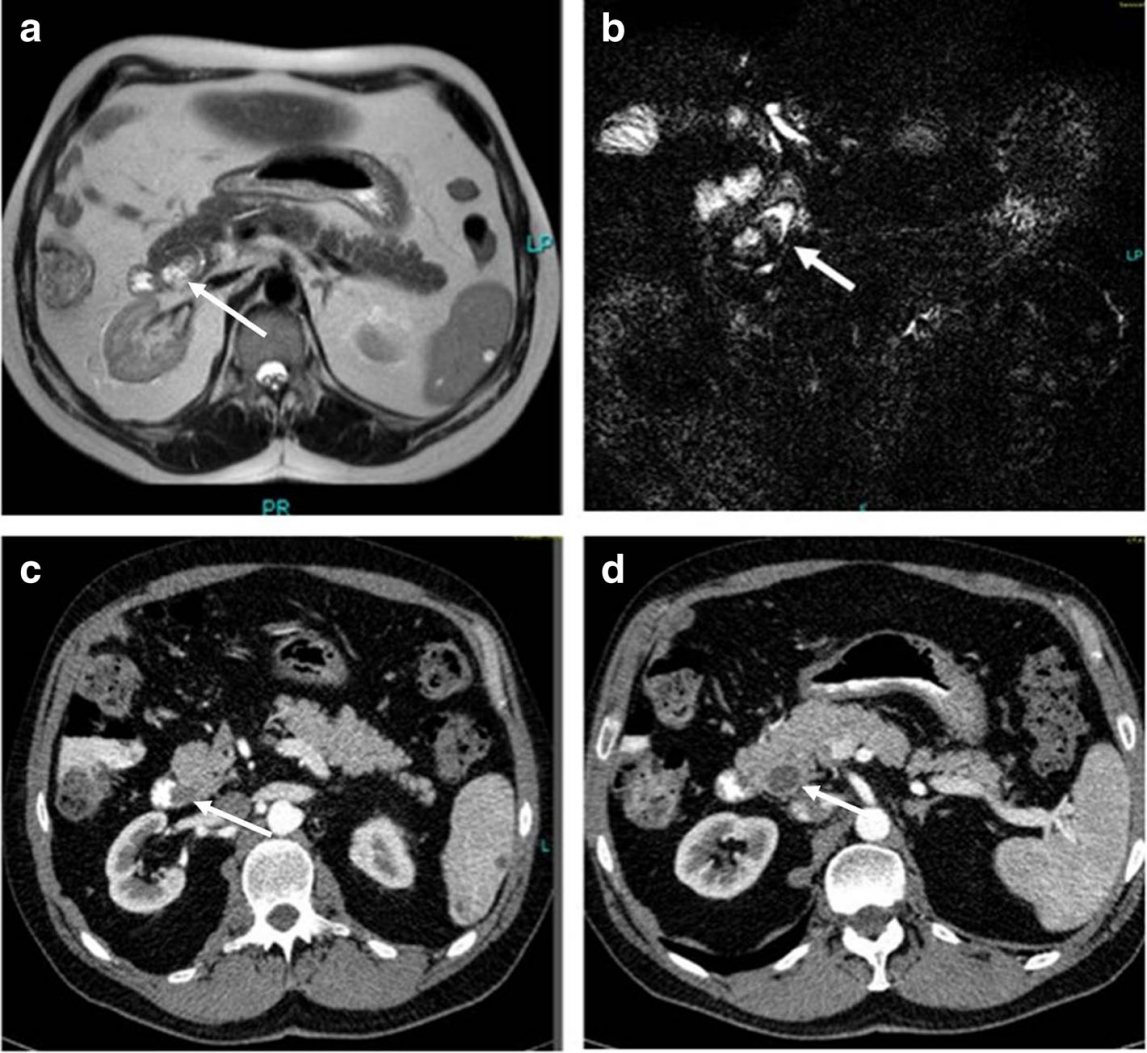
**a** Magnetic resonance imaging scan showing a contrast-enhanced mass in the pancreatic head (*arrow*). **b** Magnetic resonance cholangiopancreatography showing signal loss of the bile duct (shown by *white arrow*). **c** Computed tomography scan showing a mass near the papilla of Vater (*arrow*). **d** Computed tomography scan showing the dilation of the residual main bile duct (*arrow*)

Contrast sonography showed a hypertrophia of his left liver lobe with status post-right hemihepatectomy and no pathological findings. Computed tomography showed a dilatation of the residual common bile duct and normal size pancreatic duct and a contrast enhancement next to the papilla of Vater leading to the suspicion of an intraductal tumor. There were no signs of metastasis (Fig. 2).

On macroscopic examination, an ERCP supported the suspicion of an adenocarcinoma in his distal residual common bile duct. The pathology of the biopsy came back positive for an adenocarcinoma. An analysis of serum tumor markers (CA 19-9, CA 72-4, CA 125, and CEA) showed no elevation.

#### Treatment

He was treated with a pylorus-preserving resection of his pancreas head and his duodenum. Postoperatively, he recovered well. Oral nutrition with low amounts of fluids were started on day 1 and escalated up to solid food on day 5. He was discharged on day 14. A 6 French pancreatic drain that was placed intraoperatively was removed on day 30 during an ambulatory visit.

In addition, he received adjuvant chemotherapy with gemcitabine (1000 mg/m^2^ at day 1, 8, and 15 every 4 weeks) as monotherapy. Chemotherapy was planned for 6 months but discontinued after 3 months due to head aches and weight loss. As of April 2016, there are no signs of tumor recurrence during follow-up.

#### Pathology

A pancreatoduodenectomy was performed and on macroscopic exploration there was a 1×1×1 cm tumor at the ampullary region involving his common bile duct. Histologic examination revealed a tubular and papillary growth pattern (Fig. 1). The tumor was similar to the tumor described in his case of pCCA. On immunohistochemistry, the tumor cells expressed cytokeratin (CK) 7 and CA 19-9. The resection margins were free and the tumor was staged as pT2 pN0 (0/14) R0, G2.

#### Molecular pathology

Targeted next-generation sequencing (NGS) was performed on formalin-fixed paraffin-embedded (FFPE) samples of both cases as described before [13]. Isolated DNA (<0.5 to 97.6 ng/μl) was amplified with an in-house specified, customized Ion AmpliSeq Primer Pool. The panel comprises amplicons of 12 different genes. Polymerase chain reaction (PCR) products were ligated to adapters and enriched for target regions using the Ion AmpliSeq Panel™ Library kit according to the manufacturer’s instructions (Life Technologies). The generated libraries were equimolar pooled for amplicon sequencing to a concentration of 20 nM of each sample to counterbalance differences in sample quality. Sequencing was performed on an Illumina MiSeq benchtop sequencer (Illumina, San Diego, USA). Results were visualized in the Integrative Genomics Viewer (IGV) and manually analyzed. A 5 % cutoff for variant calls was used and results were only interpreted if the coverage was >100. Both tumors were analyzed as described and the results are shown in Table 1. An identical activating mutation of the *PIK3CA* gene exon 20 was found in both tumors.

**Table 1:** Targeted next-generation sequencing of both tumors showing the same results with an identical activating mutation in the *PIK3CA* gene exon 20

Gene	Exon	Codon	Mutation	Interpretation
*BRAF*	15	582–612	Wildtype	
*DDR2*	3–18	1–342, 351–395, 432–586, 620–856	Wildtype	
*ERBB2*	8, 19–21	302–321, 761–881	Wildtype	
*HRAS*	2–4	1–35, 41–82, 105–150	Wildtype	
*KEAP1*	2–6	1–34, 37–122, 127–437, 443–625	Wildtype	
*KRAS*	2–4	4–66, 111–150	Wildtype	
*NFE2L2*	2	16–104	Wildtype	
*NRAS*	2–4	1–32, 45–83, 110–150	Wildtype	
*PIK3CA*	1, 4, 7, 9, 20	66–117, 312–350, 418–435, 521–554, 980–1069	EX20: c.3140A>G p.H1047R	activating
*PTEN*	1–9	1–34, 39–267, 275–333, 343–366	Wildtype	
*RHOA*	2, 3	3–89	Wildtype	
*TP53*	5–9	133–260, 263–331	Wildtype	

## Discussion

A literature research revealed only a few cases comparable to our case. Nakahira *et al*. [14] reported a study with two cases of lower bile duct recurrence after hepatectomy for pCCA. In contrast to our case, both patients showed a recurrence after less than 18 months [14], indicating that both recurrences derived from residual cells after the first surgery.

There are several case reports showing that intramucosal tumor residuals after resection might lead to recurrences of the tumor especially at the side of anastomosis [15–17]. A case report from Thomas and Heaton [12] shows that even after 8 years a recurrence of the tumor is possible. In contrast to our case, their patient presented with metastatic disease and no primary was found [12].

Jang *et al*. [18] evaluated the outcome after surgical resection for extrahepatic CCA. Of the treated patients, 29 % survived 5 years without recurrences of the disease. At 5 years, 3, 4 % of the treated patients had a recurrence of the cancer with only one patient presenting with a limited disease acceptable for surgery.

The case presented here shows an example of a patient developing a secondary tumor in the same environment but anatomically distant 9 years after the primary diagnosis of pCCA. The identical mutation pattern and the absence of the most frequent gene alterations [19] in both tumors indicate that the second tumor is very likely a recurrence of the first. One reason for the long disease-free interval could be the tumor genetics. Neither a *KRAS* mutation nor a *TP53* mutation was detected, which are both linked to a worse outcome [19].

With the ongoing development of phosphoinositide 3-kinase (PI3Kinase)-specific inhibitors as targeted therapy for tumors with activating mutations in the *PIK3CA* gene as in our case, further treatment options might be available in the future [20].

## Conclusion

Late and far distant recurrence of CCA in long-time survivors is possible and could be caused by a special tumor biology.

## References

[1] Rizvi S, Gores GJ. Pathogenesis, diagnosis, and management of cholangiocarcinoma. Gastroenterology. 2013;145(6):1215–29. 10.1053/j.gastro.2013.10.013 PMC3862291 24140396

[2] Everhart JE, Ruhl CE. Burden of digestive diseases in the United States part I: overall and upper gastrointestinal diseases. Gastroenterology. 2009;136(2):376–86. 10.1053/j.gastro.2008.12.015 19124023

[3] Tyson GL, El-Serag HB. Risk factors for cholangiocarcinoma. Hepatology. 2011;54(1):173–84. 10.1002/hep.24351 PMC3125451 21488076

[4] Blechacz B, Komuta M, Roskams T, Gores GJ. Clinical diagnosis and staging of cholangiocarcinoma. Nat Rev Gastroenterol Hepatol. 2011;8(9):512–22. 10.1038/nrgastro.2011.131 PMC3331791 21808282

[5] Deoliveira ML, Schulick RD, Nimura Y, Rosen C, Gores G, Neuhaus P, et al. New staging system and a registry for perihilar cholangiocarcinoma. Hepatology. 2011;53(4):1363–71. 10.1002/hep.24227 21480336

[6] Shaib YH, El-Serag HB, Davila JA, Morgan R, McGlynn KA. Risk factors of intrahepatic cholangiocarcinoma in the United States: a case-control study. Gastroenterology. 2005;128(3):620–6. 10.1053/j.gastro.2004.12.048 15765398

[7] Welzel TM, Graubard BI, El-Serag HB, Shaib YH, Hsing AW, Davila JA, et al. Risk factors for intrahepatic and extrahepatic cholangiocarcinoma in the United States: a population-based case-control study. Clin Gastroenterol Hepatol. 2007;5(10):1221–8. 10.1016/j.cgh.2007.05.020 PMC2083573 17689296

[8] Mosconi S, Beretta GD, Labianca R, Zampino MG, Gatta G, Heinemann V. Cholangiocarcinoma. Crit Rev Oncol Hematol. 2009;69(3):259–70. 10.1016/j.critrevonc.2008.09.008 18977670

[9] Shaib YH, Davila JA, McGlynn K, El-Serag HB. Rising incidence of intrahepatic cholangiocarcinoma in the United States: a true increase? J Hepatol. 2004;40(3):472–7. 10.1016/j.jhep.2003.11.030 15123362

[10] Khan SA, Thomas HC, Davidson BR, Taylor-Robinson SD. Cholangiocarcinoma. Lancet. 2005;366(9493):1303–14. 10.1016/S0140-6736(05)67530-7 16214602

[11] Wakai T, Shirai Y, Moroda T, Yokoyama N, Hatakeyama K. Impact of ductal resection margin status on long-term survival in patients undergoing resection for extrahepatic cholangiocarcinoma. Cancer. 2005;103(6):1210–6. 10.1002/cncr.20906 15685618

[12] Thomas H, Heaton ND. Late recurrence after surgery for cholangiocarcinoma: implications for follow-up? Hepatobiliary Pancreat Dis Int. 2008;7(5):544–6. 18842505

[13] Ihle MA, Fassunke J, Konig K, Grunewald I, Schlaak M, Kreuzberg N, et al. Comparison of high resolution melting analysis, pyrosequencing, next generation sequencing and immunohistochemistry to conventional Sanger sequencing for the detection of p.V600E and non-p.V600E *BRAF* mutations. BMC Cancer. 2014;14:13. 10.1186/1471-2407-14-13 PMC3893431 24410877

[14] Nakahira S, Takeda Y, Kawashima H, Mukai Y, Hamanaka M, Uchiyama C, et al. Two cases of lower bile duct recurrence resected by pancreatoduodenectomy after hepatectomy for hilar cholangiocarcinoma. Gan To Kagaku Ryoho Cancer Chemother. 2012;39(12):2119–21. 23267996

[15] Nakanishi Y, Kondo S, Hirano S, Ambo Y, Tanaka E, Morikawa T, et al. Recurrence of mucosal carcinoma of the bile duct, with superficial flat spread, 12 years after operation. J Hepatobiliary Pancreat Surg. 2006;13(4):355–8. 10.1007/s00534-006-1092-6 16858550

[16] Sasaki T, Kondo S, Ambo Y, Hirano S, Sichinohe T, Kaga K, et al. Local recurrence at hepaticojejunostomy 9 years after resection of bile duct cancer with superficial flat spread. J Hepatobiliary Pancreat Surg. 2006;13(5):458–62. 10.1007/s00534-005-1096-7 17013723

[17] Tanaka N, Nobori M, Kohzuma T, Suzuki Y, Saiki S. Anastomotic recurrence at hepaticojejunostomy in a long-term survivor of bile duct carcinoma: report of a case. Surg Today. 1994;24(3):280–4. 10.1007/BF02032903 8003873

[18] Jang JY, Kim SW, Park DJ, Ahn YJ, Yoon YS, Choi MG, et al. Actual long-term outcome of extrahepatic bile duct cancer after surgical resection. Ann Surg. 2005;241(1):77–84. 10.1097/01.sla.0000150166.94732.88 PMC1356849 15621994

[19] Churi CR, Shroff R, Wang Y, Rashid A, Kang HC, Weatherly J, et al. Mutation profiling in cholangiocarcinoma: prognostic and therapeutic implications. PLoS One. 2014;9(12):e115383. 10.1371/journal.pone.0115383 PMC4275227 25536104

[20] Deshpande V, Nduaguba A, Zimmerman SM, Kehoe SM, Macconaill LE, Lauwers GY, et al. Mutational profiling reveals *PIK3CA* mutations in gallbladder carcinoma. BMC Cancer. 2011;11:60. 10.1186/1471-2407-11-60 PMC3045359 21303542

